# Lower extremity fatigue increases complexity of postural control during a single-legged stance

**DOI:** 10.1186/1743-0003-8-43

**Published:** 2011-08-04

**Authors:** Stephen J McGregor, W Jeffrey Armstrong, James A Yaggie, Erik M Bollt, Rana Parshad, Jerry J Bailey, Sean M Johnson, Aleta M Goin, Samuel R Kelly

**Affiliations:** 1 School of HPHP, Eastern Michigan University, Ypsilanti, MI, USA; 2 Division of HPE, Western Oregon University, Monmouth, OR, USA; 3 College of Health Professions, University of Findlay, Findlay, OH, USA; 4 Department of Mathematics & Computer Science, Clarkson University, Potsdam, NY, USA

## Abstract

**Background:**

Non-linear approaches to assessment of postural control can provide insight that compliment linear approaches. Control entropy (CE) is a recently developed statistical tool from non-linear dynamical systems used to assess the complexity of non-stationary signals. We have previously used CE of high resolution accelerometry in running to show decreased complexity with exhaustive exercise. The purpose of this study was to determine if complexity of postural control decreases following fatiguing exercise using CE.

**Methods:**

Ten subjects (5 M/5 F; 25 ± 3 yr; 169.4 ± 11.7 cm; 79.0 ± 16.9 kg) consented to participation approved by Western Oregon University IRB and completed two trials separated by 2-7 days. Trials consisted of two single-legged balance tests separated by two Wingate anaerobic tests (WAnT; PreFat/PostFat), or rest period (PreRest/PostRest). Balance tests consisted of a series of five single-legged stances, separated by 30 s rest, performed while standing on the dominant leg for 15-s with the participant crossing the arms over the chest and flexing the non-dominant knee to 90 degrees. High resolution accelerometers (HRA) were fixed superficial to L3/L4 at the approximate center of mass (COM). Triaxial signals from the HRA were streamed in real time at 625 Hz. COM accelerations were recorded in g's for vertical (VT), medial/lateral (ML), and anterior/posterior (AP) axes. A newly developed statistic (R-test) was applied to group response shapes generated by Karhunen Loeve (KL) transform modes resulting from Control Entropy (CE) analysis.

**Results:**

R-tests showed a significant mean vector difference (*p *< .05) within conditions, between axes in all cases, except PostFat, indicating the shape of the complexity response was different in these cases. R-test between conditions, within axis, differences were only present in PostFat for AP vs. PreFat (*p *< .05). T-tests showed a significantly higher overall CE PostFat in VT and ML compared to PreFat and PostRest (*p *< .0001). PostFat CE was also higher than PostRest in AP (*p *< .0001).

**Conclusions:**

These data indicate that fatiguing exercise eliminates the differential complexity response between axes, but increases complexity in all axes compared to the non-fatigued condition. This has implications with regard to the effects of fatigue on strategies of the control system to maintain postural control.

## Background

Balance, or postural stability, is the net result of the forces acting on the body's center-of-mass (COM) within the base of support. Blaszczyk and co-workers (1994) state that the ranges of the postural limits define the perimeters of stability and represent the maximum amount of excursion the COM may incur without falling. Impairment of the musculoskeletal and sensory systems involved in postural control is of clinical importance when characterizing the outcomes and precautions for the avoidance of traumatic injury and fall. A clinically/physiologically relevant way that postural control can be impaired is through fatiguing exercise [1,2]. Yaggie and McGregor [1] observed that fatigue of the ankle plantar and dorsi-flexors resulted in significant, but transient, changes in sway parameters and ranges of postural control. Because impaired postural control may have implications for subsequent traumatic injury in sport and recreation [3], it is important to characterize how fatigue induced in different muscle groups, or via different modalities affects the nature of impairments to postural control. In the case of the Yaggie and McGregor [1] study, the fatigue was induced through isokinetic exercise localized to small muscle groups primarily acting on the ankle joint (e.g. ankle dorsi and plantarflexors). Isokinetic exercise is not common in athletic or recreational endeavors. Further, many exercise modalities involve larger muscle groups that may be acting on joints "upstream" from the ankle joint. Therefore, it is of interest to examine the impact of more dynamic exercise affecting larger, more disparate muscle groups involved in postural control.

Multiple tools have been employed to assess balance and posture. Forceplates have been used to assess movement of the center-of-foot-pressure (COFP). According to Adlerton and others [4], this approach may have limited sensitivity in capturing the subtle changes associated with postural control as COFP provides only the summation of control mechanisms and provides no information pertinent to the discrete muscle actions that lead to postural control. Trunk accelerometry using a high-resolution accelerometer (HRA) mounted on the body at the approximate center of mass (COM) can provide an alternative or complement to forceplates in balance studies [4-6]. Accepting that the body moves as an inverted pendulum in erect posture, COM accelerations/velocities reflect postural sway, and thereby provide a useful compliment or alternative to COFP. This methodology has been supported by Moe-Nilssen and Helbostad [6] and has been demonstrated to be reliable [5]. Further, since HRA of COM measures the actual movement of the approximate COM, it can be argued this is a more reflective measure of the intended output (posture) of the controller than COP measures using forecplates, which are effectively weighted averages of forces applied diffusely over the contact surface being measured.

Typically, postural control is evaluated using traditional linear analytical approaches. Recently though, there has been increasing interest in the use of non-linear analytical techniques derived from the field of dynamical systems [7]. In particular, the use of complexity/regularity statistics such as Approximate and Sample Entropy have provided new insight into the nature of postural control, and its impairment due to traumatic brain injury [3]. A major limitation to the use of most non-linear approaches, though, is the requirement of stationarity, which limits the utility of these tools under dynamic conditions. Recently, Bollt et al. [8] have developed a novel approach to complexity analysis termed Control Entropy, which, importantly, alleviates the requirement of stationarity. This tool has been used to demonstrate distinctive constraints between different planes of movement in runners [9] as well as between groups of trained versus untrained runners [10]. As there are numerous differences between Control Entropy (CE) and other complexity statistics (e.g. Approximate Entropy; ApEn, [8], and CE is more appropriate for use under dynamic conditions, CE may provide additional insight regarding impairment of postural control that can complement information that is already available.

The purposes of the present study were to evaluate the effect of dynamic, large muscle mass fatiguing exercise on i) changes in COM accelerations and ii) the complexity of these signals as assessed with CE. To achieve these aims, we used a standard bicycle exercise protocol, the Wingate Anaerobic Test (WAnT), which is objectively quantifiable and well characterized with regard the nature of the fatigue it imparts on subjects. Further, we applied a recently developed, novel statistical approach, termed the *R*-test [10], for the rigorous comparison of within group and between group CE measures. We hypothesized that after fatiguing large muscle mass exercise of the lower limbs that i) COM accelerations would be increased relative to the control condition and ii) CE of the HRA signal would decline indicating reduced complexity and/or increased constraints of postural control.

## Methods

### Subjects

Ten participants (5 males and 5 females; mean age = 25 ± 3 yr; height = 169.4 ± 11.7 cm; weight = 79.0 ± 16.9 kg) were recruited, and consented to participation through completion of the Western Oregon University IRB approved informed consent documentation in accordance with the Declaration of Helsinki. All participants indicated by self-reported medical history that they had no known physiological or functional conditions that would prohibit them from performing exhaustive exercise for a brief period of time, and had no known, recent, or previous injuries that would prevent them from participating.

The participants reported to the Exercise Physiology Laboratory having at least 2-hours rest from exercise and 12-hour abstention from alcohol, caffeine, and any medication that affects the central nervous system. Testing days were separated by no more than seven days.

### One-legged Stances

A series of five single-legged stances were repeated twice during each of two testing days to record HRA measures for the purposes of assessing balance. Thus, four sets of five stances were grouped as follows: Pre-Rest (PreRest), Post-Rest (PostRest), Pre-Fatigue (PreFat), and Post-Fatigue (PostFat). Stances were performed while standing on the dominant leg (determined by the leg with which the participant would instinctively kick a ball) for 15-seconds with the participant crossing the arms over the chest and flexing the non-dominant knee to 90 degrees. Each stance in a set was separated by a 30-s rest period.

### COM Acceleration

A wireless HRA (G-Link, ± 10 g, MicroStrain, Inc., Williston, VT) was fixed with two-sided tape at the intersection of the sagittal and axial planes on the posterior trunk superficial to L3/L4, at the approximate center of mass [5] and secured with elastic tape (PowerFlex, Andover, MA). Triaxial signals from the HRA were streamed in real time to a base station at a frequency of 625 Hz and then exported to AcqKnowledge 4.0 (Biopac Systems, Inc., Santa Barbara, CA) for analysis. COM accelerations were recorded in g's for vertical (VT), medial/lateral (ML), and anterior/posterior (AP) axes. Reliability of these procedures has been demonstrated to be moderately strong (*r *= 0.63-0.89) and is discussed in a previous publication [11].

### Statistical Analysis

RM-ANOVAs were used to analyze the effects of fatigue. Bonferroni post hoc analyses were subsequently performed to determine group differences where applicable. All linear statistical analyses were performed using PASW statistical software v. 17.0 (SPSS Inc., α ≤ 0.05). Control Entropy, K-L analysis and R tests were performed using Matlab (The Mathworks, MA; α ≤ 0.05).

### Control entropy and Statistical Testing

From an information theory standpoint, the Shannon entropy [2], is defined as(1)

where p_i _is the probability of being in a state i. Many variants of this are actively used in the dynamical systems literature [12-14]. Recently much attention has been drawn to the approximate entropy (AE) of Pincus [15,16]. An essential requirement of this method, is an inherent assumption of stationarity. In [8] we developed a regularity statistic termed control entropy (CE). This is an entropy-like statistic, that could apply to non-stationary time series data. Non-stationarity is observed in a large number of real world processes, and thus requires the usage of a tool such as (CE). Furthermore part of our goal was to understand parameter changes within the system as a way of detecting developing problems, or to serve as a warning before system failure. The (CE) tool is well suited for this. We define the control entropy of the signal,(2)

We adopt the SAX method here and b is chosen to consist of n symbols, and xi is mapped to si according to an equipartition of Z-values from a normal model on the data set. We shall use the SAX symbolization in computing CE according to Eq. (2), where n will be chosen to satisfy the saturation criterion which we described in [8].

Our current goal is to adopt a formal statistical approach to continue the agenda of [8]. We would now like to construct something stronger than the "ellipsoid" approach, which is essentially a proper orthogonal decomposition of the CE signal, and then consideration of the first two modes. Thus we want to decide with a statistical confidence, how different two groups of balance conditions might be. We will resort to multivariate statistical analysis as we are considering the first two modes. Under the assumption of normality, we are dealing with a projective data cloud, and choose to use the Hotelling's *T*^2 ^test, [5]. This is a multivariate version of the students *t *test. The students t distribution is a continuous probability distribution that arises when one wants to estimate the mean of a normally distributed population. It is used when the sample size is small, [17]. In the multivariate setting we have vector observations, as a result of the POD routine applied to the CE signal of the raw data from the subjects.(3)

Here X_1i _represents a particular subject in say the first group with x_11 _and x_12 _the first two modes. Similarly there are X_2i_, X_3i_, ..X_ni _and Y_2i_, Y_3i_.....X_ni _for the two different groups under consideration. Thus, we have that μ_1 _is the population mean vector for the first group and μ_2 _is the population mean vector for the second group. We are interested in testing the null hypothesis that the population mean vectors for the two groups of subjects are equal, against the alternative hypothesis that these mean vectors are not equal. This can be carried out via the following procedure. Under the null hypothesis the two mean vectors are equal element by element. Thus we will look at the differences between the observations. We define(4)

We also define the vector(5)

Thus, we have now converted our original problem into a problem of testing the null hypothesis that the population mean vector μ_z _= 0. This hypothesis is tested using the paired Hotelling's *T*^2 ^test. We reject the null hypothesis at level α if the F-value exceeds the value with p and n-p degrees of freedom, evaluated at level α, which for our purposes (as well as in most cases) is set at 0.05. The computations for the above were carried out in MATLAB. We developed code to symbolize the raw data, from which the CE is calculated. This is passed into a second routine which performs the POD, and yields the dominant modes, for subjects for the groups in question. This is finally passed pair wise, into a routine that carries out the multivariate Hotelling *T^2^*test, yielding the statistics of interest, which essentially allows us to compare the groups. For details the reader is referred to [10].

## Results

Mean peak-peak amplitudes for HRA are presented in Table 1. RM-ANOVA revealed no significant effects for time × session × axis (*p *= 0.99) and time × axis (*p = *0.40). There were significant effects for time, session, and time × session (*p *< 0.001). Post hoc analyses confirmed no differences between resting measures and a significant effect of fatigue (*p *≤ 0.003).

**Table 1:** Mean peak-peak amplitudes for and HRA (g).

	PreRest	PostRest	PreFat	PostFat
**Vertical (VT)**	0.074 ± 0.026	0.069 ± 0.022	0.074 ± 0.025	0.128 ± 0.050*
**Medial-Lateral (ML)**	0.101 ± 0.038	0.098 ± 0.033	0.102 ± 0.038	0.153 ± 0.650*
**Anterior-Posterior (AP)**	0.074 ± 0.020	0.073 ± 0.023	0.071 ± 0.026	0.099 ± 0.032*
**Resultant (RES)**	0.072 ± 0.025	0.067 ± 0.020	0.089 ± 0.066	0.187 ± 0.251*

*significantly greater than resting conditions (PreRest, PostRest, and PreFat), *p *≤ 0.003.

### Control Entropy of HRA: Mean Vector Differences (R-test) between Axes

Results of the *R*-test for PreFat condition between axes can be found in Table 2. Mean vector differences were significant between all axes within this condition. Similarly, the PostRest condition resulted in significant Mean Vector differences between all axes, and the PreRest Mean Vector differences were significant between VT vs ML and ML vs AP, and a trend (*p *= 0.11) for VT vs AP was present. No Mean Vector differences were present for the PostFat condition between axes.

**Table 2:** Within Treatment Effects.

Mean Vector Differences for CE of HRA	VT vs ML	ML vs AP	VT vs AP
**PreRest**	0.020	0.014	0.110
**PostRest**	0.004	< 0.000	0.040
**PreFat**	0.001	< 0.000	0.030
**PostFat**	0.450	0.130	0.180

Mean Differences for CE of HRA	**VT vs ML**	**ML vs AP**	**VT vs AP**

**PreRest**	NA	NA	0.914
**PostRest**	NA	NA	NA
**PreFat**	NA	NA	NA
**PostFat**	0.560	0.930	0.480

### Control Entropy of HRA: PreFat vs. PostFat by Axis

**Figure 1:**
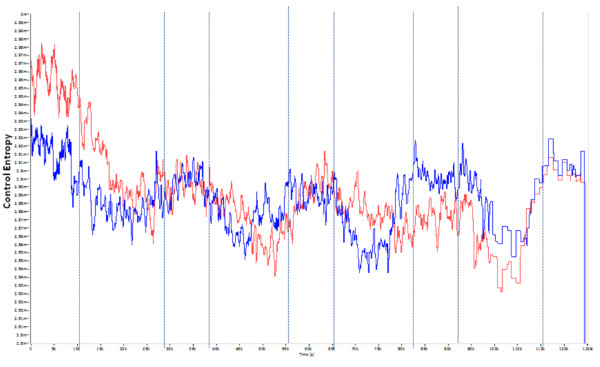
**Dominant modes of K-L analysis performed on Control Entropy outputs of HRA signal of the VT axis collected during single-legged stance for PreFat (White) and PostFat (Red)**. Shape of the dominant modes is not significantly different. Mean of PostFat significantly higher than PreFat (*p *< 0.001). Yellow lines indicate beginning and end of single legged stance tests.

Comparison of the K-L dominant modes in the VT axis for PreFat vs. PostFat can be seen in Figure 1. The *R*-test showed no statistical difference (Table 3) between shapes of the PreFat and PostFat conditions, but a *t-*test showed a significant difference (Table 3) in overall CE between conditions indicating that CE was generally lower in the PreFat condition vs. the PostFat condition in the VT axis. Further, the PostRest condition was also significantly lower than PostFat in the VT axis.

**Table 3:** Between Treatment Effects.

Mean Vector Differences for CE of HRA	VT	ML	AP
**PreRest vs. PostRest**	0.989	0.808	0.851
**PostRest vs. PostFat**	0.185	0.212	0.058
**PreFat vs. PostFat**	0.256	0.059	< 0.0000

Mean Differences for CE of HRA	**VT**	**ML**	**AP**

**PreRest vs. PostRest**	8.6^-30	9.8^-32	2.1^-27
**PostRest vs. PostFat**	3.0^-14	1.1^-21	8.3^-22
**PreFat vs. PostFat**	1.4^-232	7.1^-256	NA

In all cases of significant values for Mean Differences, PostRest was greater than PreRest and Post Fat was greater than Post Rest and PreFat

**Figure 2:**
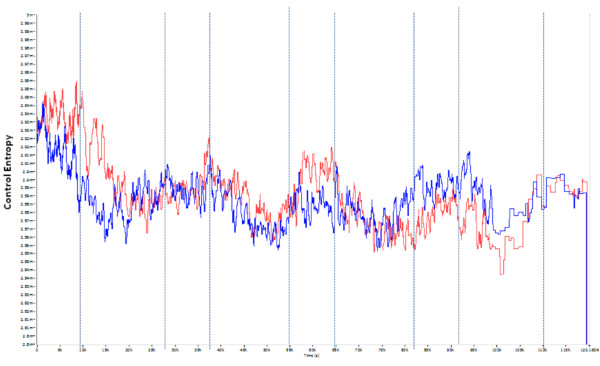
**Dominant modes of K-L analysis performed on Control Entropy outputs of HRA signal of the ML axis collected during single-legged stance for PreFat (White) and PostFat (Red)**. Shape of the dominant modes is not significantly different (*p *= 0.059). Mean of PostFat significantly higher than PreFat (*p *< 0.001). Yellow lines indicate beginning and end of single legged stance tests.

Comparison of the K-L dominant modes in the ML axis for PreFat vs. PostFat can be seen in Figure 2. The *R*-test showed no statistical difference, but a trend (Table 3) between shapes of the PreFat and PostFat conditions. Also, a *t*-test showed a significant difference (Table 3) in overall CE between conditions indicating that CE was generally lower in the PreFat condition vs. the PostFat condition in the ML axis. As with the VT axis, the PostRest condition was also significantly lower than PostFat in the ML axis.

**Figure 3:**
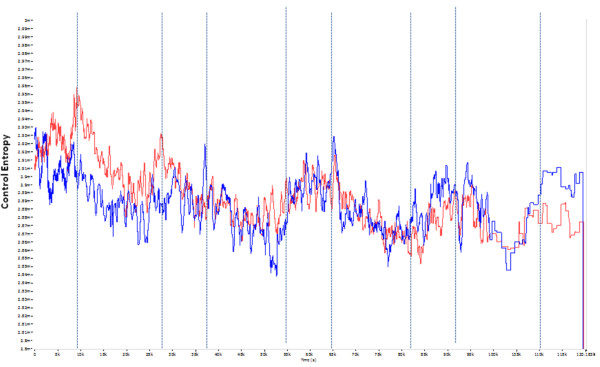
**Dominant modes of K-L analysis performed on Control Entropy outputs of HRA signal of the AP axis collected during single-legged stance for PreFat (White) and PostFat (Red)**. Shape of the dominant modes is significantly different (*p *< 0.001). Yellow lines indicate beginning and end of single legged stance tests.

Comparison of the K-L dominant modes in the AP axis for PreFat vs. PostFat can be seen in Figure 3. The *R*-test showed a highly significant difference, (Table 3) between shapes of the PreFat and PostFat conditions. Because of the significantly different shape of the dominant modes in this axis, it was not appropriate to perform a *t*-test to quantify differences in absolute CE values between the two conditions. No significant difference was observed between the shapes of PostRest and PostFat by virtue of the *R*-Test, therefore a *t*-test was performed and CE of the PostRest condition was generally lower than the PostFat condition in the AP axis.

## Discussion

In this study, we present a novel approach to investigate the effects of lower limb fatigue on both the linear measures, as well as the complexity (i.e. regularity) of postural control. Complexity was evaluated through assessment of Control Entropy of COM- HRA signal, and a novel statistical approach was used for this purpose. From a linear perspective, it was hypothesized that fatiguing lower limb exercise would result in greater COM accelerations during one-legged stance compared to the control condition. This was indeed the case as there was a significant effect for fatigue on COM accelerations during one-legged stance. With regard to non-linear aspects of the study, it was hypothesized that fatiguing lower limb exercise would increase the constraints on the components of control of posture, as evidenced by reduced CE. Contrary to this hypothesis, changes in CE with fatiguing exercise indicated increased complexity of postural control. The implications of this novel insight are discussed herein.

### Fatigue and linear characteristics of postural control

The effects of fatigue on the maintenance of postural control mechanisms have been well documented [1,4,6,18]. Previously, we have reported differences in sway parameters and balance indices immediately post-fatigue utilizing both generalized lower extremity exercise (WanTs) [18], as well as localized ankle isokinetics [1]. Further, Alderton, Moritz and Moe-Nilssen [4] investigated the relationship between forceplate and tri-axial accelerometer measures of the one-legged stance pre- and post-fatigue. Force plate analysis of center-of-pressure velocity and amplitude in the M/L and A/P were observed, while similar directional observations were recorded using a tri-axial accelerometer. The results showed significant increase in trunk acceleration and center-of-pressure amplitude in both directions and a significant decrease in center-of-pressure velocity in both directions, indicating that the foot may have been in a more secure posture, while the trunk oscillated to stabilize the perturbations in stance. Further analysis concluded that there was a moderate correlation between force plate center-of-pressure measurements and trunk accelerometer accelerations. The investigators concluded that trunk accelerometry may be a more appropriate measure for changes occurring at the hip and trunk and may provide an alternate facet of kinematic analysis that kinetics may not detect. In the present study, RM-MANOVA revealed differences in all of linear HRA values (AP, VT, ML, RES), post-fatigue, and the directional analyses are consistent with the previous literature [4].

Linear changes in AP values were slight but significant (Table 1). A possible explanation for these changes includes the fact that under the fatigued condition, the anterior orientation remains quite stable, likely due to the ability of the visual system to attenuate mechanisms of fatigue via visual awareness of self-to-object orientation. Further, the anterior projection of the forefoot allows for a semi-rigid lever for steadiness and segmental correction. It is likely that the differences in the linear AP values are attributed to the more posterior projection of the COM in maintenance of posture post-fatigue, and may also relate to the changes in vertical accelerations observed, post exercise. As the musculature of the lower extremity becomes unresponsive in the sensory and motor domains under fatigue, it relies more heavily on the unfatigued trunk musculature to make a rapid correction in stance. The increase in AP and VT values are likely due to an increase in trunk extension intended to mediate the alterations in body sway by aligning the COM in a more erect posture. Spinal extension represents a conjugate motion in both the posterior and vertically positive direction, supporting the present observations.

ML corrections were also notable in the post-fatigue condition. The significant increases in linear ML values are consistent with the literature [4]. As the body begins to sway post-fatigue, the small musculature of the lateral leg compartment (peroneal muscles) fails to respond to the demands placed on it by the COM excursion. Therefore, the corrections are typically made more superiorly in the kinetic chain at the level of the trunk. Again, the unfatigued trunk musculature may contract to more effectively correct posture and maintain stance.

The resultant acceleration of the COM, derived from the HRA values, will represent the resolved vector for all motion about the trunk noted in this condition. Each axes of motion exhibited an increase in acceleration following the WAnT trials. The linear RES HRA values, in-turn, exhibited significant alteration post-fatigue. These values provide a resolution of all directions of motion observed over time. It is not surprising that the significantly positive resolution of the AP, VT and ML linear HRA values exhibited in the RES measure would represent an increase in trunk (COM) excursion post-fatigue.

### Fatigue and complexity of postural control

**Figure 4:**
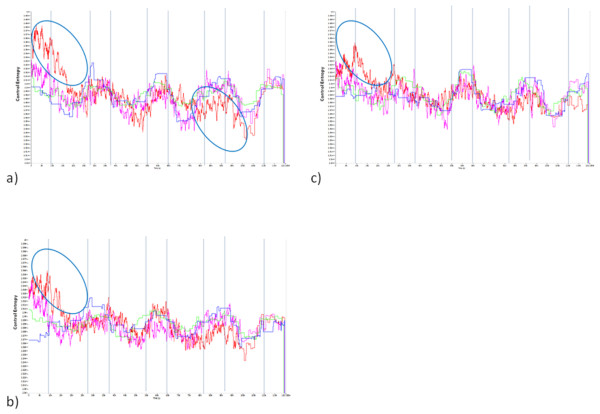
**Dominant modes of K-L analysis performed on Control Entropy outputs of HRA signal of the a) VT, b) ML and c) AP axes collected during single-legged stance for PreRest (Blue), PostRest (Green) PreFat (White) and PostFat (Red)**. Yellow circles indicate segments where PostFat CE is apparently higher than in all other conditions.

Although much of the linear HRA responses were anticipated, and agreed with the literature or stated hypotheses; in contrast, it was not anticipated that fatiguing lower limb exercise would elicit an increase in CE of COM accelerations. In general, relatively high entropy indicates high complexity/low constraints, and on the other hand, low entropy indicates low complexity/high constraints [8]. In the current work, we hypothesized that fatiguing exercise of the lower limbs would result in increased constraints imposed upon postural control, and this would be evidenced by reduced CE of HRA signal in the PostFat condition, but this was not the case. In all conditions where comparison of mean CE between conditions was appropriate, CE of the fatigued condition (PostFat) was higher than either of the non-fatigued conditions (PreFat and PostRest; Table 3). Further, in the single case (AP; Table 3) where an R-test indicated a different shape of the CE response was elicited by the fatigued condition (PostFat) relative to the non-fatigued condition (PreFat), it appears as though the CE is higher during fatigue than all other conditions (Figures 3 and 4). If the shapes of the dominant modes are not different, then it follows that the time evolutions of the dynamical characteristics of the signals are not different and this may be appropriately tested by a simple means comparison between two conditions. On the other hand, if there is a significant difference in the shape of dominant modes, the conclusion is that the time evolution of the dynamical characteristics of the signal are significantly different. Comparison of means between the two conditions should be viewed with caution, and may be entirely inappropriate.

The hypothesis that fatigue would elicit a reduction in CE of HRA during balance tasks was stimulated by 1) observations that Approximate Entropy (ApEn) of center of pressure oscillations is reduced after concussion [19] and 2) previous data using CE of HRA signal in running where CE was reduced dramatically with fatigue in all axes [9]. Since it had previously been reported that complexity of postural control was reduced with concussion (increased constraint) and we had observed decreased CE of HRA with exhaustion in running, it followed that with exhaustion/fatigue of the lower limbs with cycling, we would see reduced CE of signal associated with postural control. This was not the case, but it is not without precedent. Cavanaugh has also reported using ApEn that complexity of balance increased with the addition of a cognitive task [20], and Donker has also reported that regularity of postural control is reduced (complexity increased) with the addition of a cognitive task [7]. Therefore, it may be that in the case of single leg balance and quiet stance, the induction of a fatigued state of the muscles of the lower limb is more akin to adding a cognitive task than to impaired postural control resulting from acute brain injury. In particular with regard to the Cavanaugh study, it is interesting to note that with the addition of the cognitive task, complexity only changed in the AP axis, and not the ML. In the current study, although we used a different approach, that of the R-test of K-L transformations, the only PostFat value that was different from PreFat was the AP value, indicating a differential shape of the CE response. Despite this, there were strong trends for different shapes of the K-L transforms for the ML axis compared to PreFat, and for the AP axis compared to PostRest (control). So, it appears as though the AP axis is most susceptible to changes in the shape of the CE response with fatigue. Despite this, all axes were significantly higher for PostFat compared to PreFat and PostRest. Further, all axes were significantly higher in the PostRest vs. PreRest condition. This indicates that there is likely some adaptation in the short- term that results in greater complexity in postural control with one familiarization trial (PreRest), but that the increases in complexity with fatigue are over and above those increases that occur as a result of familiarization.

The increased complexity of postural control in the PostFat condition may be analogous to the condition reported by Donker et al. [7] with added "dual tasks". In the Donker work [7], adding a single cognitive task to standing with eyes closed in healthy young subjects, complexity decreased (reduced Sample Entropy), but if a second cognitive task was added, complexity increased relative to the single task condition. It should be noted that in the Donker study, postural control was assessed during two-legged standing, and the authors surmised that two-legged standing while performing dual tasks with eyes open was not challenging, but with eyes closed and performing a single task, attention was diverted to postural control and complexity declined, while adding in the second task diverted attention away from postural control, thus increasing complexity of the task. Therefore, if we accept Donker's assertion that increased complexity of postural control is indicative of reduced/diverted attention, we can interpret the current results to indicate that 1) the "learning effect" of the post-Rest condition results in reduced attention to the task of postural control and 2) the addition of fatigue results in greater diversion of attention away from postural control than the learning effect itself. The question arises then, if fatigue causes attention to be diverted away from the postural control task, to where is the attention diverted? Further work will be necessary to experimentally address this question.

It is of interest to contrast the results of the current study with previous results we have obtained performing CE analysis of HRA during exhausting run tests. Although not directly comparable with regard to the role of attention on balance, comparing these results eliminates potential confounding factors of data collection (e.g. force plates vs. HRA) and analysis (e.g. Approximate Entropy vs. Sample Entropy vs. Control Entropy). In other words, the differences in complexity/regularity found between studies using different collection methods and, particularly regularlity statistics, cannot be attributed solely to biological factors. For example, the technical differences between Approximate Entropy, Sample Entropy and Control Entropy have been addressed at length previously [8], and therefore, factors such as partition number and or data stationarity cannot be ruled out as sources of discrepancies in results between studies using these techniques. In our running work though, HRA data were collected and analyzed similarly to the current study, and therefore differences in the CE of HRA response should be attributed to the constraints imposed and the controller's efforts to address them [8]. So, the fact that CE of HRA signal declines at exhaustion during running contrasts with the increased CE of HRA signal after fatiguing exercise in the current study. Is this difference due to the differences in the nature of the tasks (i.e. single-legged balance vs. running) or due to the constraints imposed? Further work will be necessary to elucidate this question. Additionally, future work should address differences in data collection methods (i.e. force plate vs. HRA) during the balance task following fatiguing exercise. For example, performing CE analysis of force plate and HRA data collected simultaneously will resolve any discrepancies present as a result of technical differences between data collection methods, while providing additional insight regarding the differences in control constraints in two different "local" dynamical environments (i.e. COM vs COFP).

## Conclusions

We report here that fatiguing exercise of the lower limbs affects both linear and non-linear characteristics of postural control. Most notably, complexity of postural control is increased following two successive Wingate anaerobic tests. The underlying reason for this increased complexity is not clear, but may be because fatigue of lower limbs imparts an increased requirement for attention to the balance task. Alternatively, fatigued muscles may be ineffective at properly implementing the controller's output commands, hence requiring greater exploration of solutions to the postural control problem in this state. Our statistical approach allowed us to determine differences in complexity that were due simply to a learning effect as opposed to those that were due to fatigue itself. These results have clinical implications for the maintenance of postural control and the prevention of traumatic injury in the fatigued state, and provide a foundation for future work in this area.

## Competing interests

The authors declare that they have no competing interests.

## Authors' contributions

SJM, WJA, JAY participated in study design, data analysis and manuscript preparation. EMB and RP performed data analysis and contributed to manuscript preparation. JJB, SMJ, AMG and SRK participated in the design and coordination of the data collection. All authors read and approved of the final manuscript.
